# Supplementary material for: Determinants and constraints of feather growth
Source: PLoS One. 2020 Apr 24;15(4):e0231925. doi: 10.1371/journal.pone.0231925 (PMC7182269; doi:10.1371/journal.pone.0231925)
Supplement: S1 Table — (PDF) [file pone.0231925.s001.pdf]

S1 Table. Measurements of the longest primary of 27 species, ordered by body mass (taken from Cramp et al. 1977 – 1994). The column 'source' indicates where growth rates by length or growth duration were taken from. See Materials and Methods for details.

Species		Body mass (g)	Longest primary	Feather mass (mg)	Feather length (mm)	Calamus diameter dorso-ventral (mm)	Calamus diameter distal-proximal (mm)	Calamus circumference (mm)	Calamus cross-sectional area (mm^2)	Duration of feather growth (d)	Sample size	Source
Willow Warbler	*Phylloscopus trochilus*	9.0	P8	7.5	61	.800	.908	2.680	.5702	20.12	4	Bensch et al. 1991
Coal Tit	*Parus ater*	9.5	P7	5.5	57	.731	.775	2.370	.4451	18.92	4	Polo & Carrascal 1999
Bluethroat	*Luscinia svecica*	18.4	P8	9.0	61	.795	.850	2.580	.5307	18.83	1	Bensch et al. 1991
Barn Swallow	*Hirundo rustica*	18.7	P9	34.0	109	1.340	1.395	4.300	1.4681	27.25	1	Saino et al. 2012, Saino et al. 2013
European white wagtail	*Motacilla alba*	21.6	P8	16.0	78	1.045	1.245	3.600	1.0218	17.33	1	Persson 1977
Common Wheatear	*Oenanthe oenanthe*	22.9	P8	19.0	82	1.280	1.315	4.080	1.3220	27.33	1	Williamson 1957
Eurasian Tree Sparrow	*Passer montanus*	23.0	P7	10.5	60	.800	.893	2.660	.5608	19.96	4	own data
Eurasian Bullfinch	*Pyrrhula pyrrhula*	26.0	P6	17.0	80	1.038	1.225	3.560	.9982	29.63	2	Newton 1967
European Greenfinch	*Carduelis chloris*	27.5	P8	20.0	79	1.200	1.255	3.860	1.1828	32.92	1	Newton 1967
House Sparrow	*Passer domesticus*	30.0	P8	15.0	67	.897	1.032	3.030	.7273	17.05	4	Zeidler 1966
Eurasian Skylark	*Alauda arvensis*	34.5	P8	25.0	88	1.195	1.420	4.120	1.3327	18.72	1	own data
White-winged Snowfinch	*Montifringilla nivalis*	37.0	P8	38.0	104	1.300	1.570	4.520	1.6030	25.37	1	Winkler & Winkler 1985
Common Starling	*Sturnus vulgaris*	80.0	P9	65.0	109	1.570	1.750	5.220	2.1579	27.25	1	Dawson 2003
Common Quail	*Coturnix coturnix*	110.0	P9	36.0	90	1.380	1.240	4.120	1.3440	23.94	1	Baader 1982
Eurasian Kestrel femal	*Falco tinnunculus*	180.0	P8	270.2	211	2.339	3.184	8.730	5.8550	46.14	4	Piechocki 1956
Alpine Chough	*Pyrrhocorax graculus*	210.0	P7	296.0	232	2.525	3.495	9.520	6.9310	37.42	1	Winkler et al. 1988
Magpie	*Pica pica*	210.0	P6	149.0	169	1.960	2.805	7.540	4.3180	51.21	1	Seel 1976
Long-Eared Owl	*Asio otus*	290.0	P8	393.0	251	3.260	3.300	10.300	8.4493	39.84	1	Wijnandts 1984
Common	*Perdix perdix*	380.0	P7	132.0	135	2.630	2.615	8.240	5.4015	27.22	1	Baader 1982

Partridge												
Rook	*Corvus frugilegus*	470.0	P7	553.0	272	3.005	4.045	11.130	9.5467	61.82	1	Seel 1976
Carrion Crow	*Corvus corone*	540.0	P7	623.0	283	3.560	4.315	12.400	12.0648	57.76	1	own data
Mallard	*Anas platyrhynchos*	1016.0	P9	434.0	204	3.500	3.420	10.870	9.4012	37.09	2	Panek & Majewski 1990
Common pheasant	*Phasianus colchicus*	1150.0	P7	444.0	205	3.395	4.015	11.660	10.7057	32.03	1	Sutter 1971
Common Raven	*Corvus corax*	1200.0	P7	1602.0	379	4.680	5.868	16.620	21.5669	61.13	2	own data
White Stork	*Ciconia ciconia*	3448.0	P8	2594.0	453	6.715	7.094	21.700	37.4134	52.07	4	Sutter 1984
Golden Eagle	*Aquila chrysaetos*	5194.0	P6	5432.0	571	7.300	9.300	26.170	53.3206	63.44	1	own data
Mute Swan	*Cygnus olor*	10750.0	P8	4217.0	455	7.790	7.850	24.570	48.0282	65.94	1	Mathiasson 1973

References

BAADER, E. (1982): Vergleichende Untersuchungen über Ausbildung und Mauser des Jugendgefieders bei Wachtel, Rebhuhn und weiteren Hühnervögeln. PhD thesis, University of Basel.

BENSCH, S., D. HASSELQUIST, A. HEDENSTRÖM & U. OTTOSSON (1991): Rapid moult among Palaearctic passerines in West Africa - an adaptation to the oncoming dry season? Ibis 133: 47–52.

CRAMP, S. *et al.* (1977-1994): Handbook of the Birds of Europe, the Middle East and North Africa. The Birds of the Western Palearctic, Vol. 1-9. Oxford Univ. Press, Oxford.

DAWSON, A. (2003): A detailed analysis of primary feather moult in the Common Starling *Sturnus vulgaris* - new feather mass increases at a constant rate. Ibis 145: E69-E76.

MATHIASSON, S. (1973): A moulting population of non-breeding Mute Swans with special reference to flight-feather moult, feeding ecology and habitat selection. Wildfowl 24: 43–53.

NEWTON, I. (1967): Feather growth and moult in some captive finches. Bird Study 14: 10–24.

PANEK, M. & P. MAJEWSKI (1990): Remex growth and body mass of mallards during wing molt. Auk 107: 255–259.

PERSSON, C. (1977): The early stages of the postnuptial moult in the White Wagtail *Motacilla alba*. Ornis Scandinavica 8: 97–99.

PIECHOCKI, R. (1956): Über die Mauser eines gekäfigten Turmfalken (*Falco tinnunculus*). J. Ornithol. 97: 301–309.

POLO, V. & L. M. CARRASCAL (1999): Ptilochronology and fluctuating asymmetry in tail and wing feathers in Coal Tits *Parus ater*. Ardeola 46: 195–204.

SAINO, N., M. ROMANO, M. CAPRIOLI, R. AMBROSINI, D. RUBOLINI, C. SCANDOLARA & A. ROMANO (2012): A ptilochronological study of carry-over effects of conditions during wintering on breeding performance in the barn swallow Hirundo rustica. J. Avian Biol. 43: 513–524.

SAINO, N., M. ROMANO, M. CAPRIOLI, R. LARDELLI, P. MICHELONI, C. SCANDOLARA, D. RUBOLINI & M. FASOLA (2013): Molt, feather growth rate and body condition of male and female Barn Swallows. J. Ornithol. 154: 537–547.

SEEL, D. C. (1976): Moult in five species of corvidae in Britain. Ibis 118: 491–536.

SUTTER, E. (1971): Ausbildung und Mauser des Flügelgefieders beim juvenilen Jagdfasan *Phasanius colchicus*. Ornithol. Beob. 68: 179–222.

SUTTER, E. (1984): Ontogeny of the wing moult pattern in the White Stork *Ciconia ciconia*. Proceedings V Pan-African Ornithological Congress 543–551.

WIJNANDTS, H. (1984): Ecological energetics of the Long-eared Owl *Asio otus*. Ardea 72: 1–92.

WILLIAMSON, K. (1957): The annual post-nuptial molt in the wheatear *(Oenanthe oenanthe)*. Bird Banding 28: 129–135.

WINKLER, R., W. D. DAUNICHT & L. G. UNDERHILL (1988): Die Grossgefiedermauser von Alpendohle *Pyrrhocorax graculus* und Alpenkrähe *Pyrrhocorax pyrrhocorax*. Ornithol. Beob. 85: 245–259.

WINKLER, R. & A. WINKLER (1985): Zur Jugendmauser handaufgezogener Schneefinken *Montifringilla nivalis*. Ornithol. Beob. 82: 55–66.

ZEIDLER, K. (1966): Untersuchungen über Flügelbefiederung und Mauser des Haussperlings (*Passer domesticus* L.). J. Ornithol. 107: 113–153.
