# Supplementary material for: Determinants and constraints of feather growth
Source: PLoS One. 2020 Apr 24;15(4):e0231925. doi: 10.1371/journal.pone.0231925 (PMC7182269; doi:10.1371/journal.pone.0231925)
Supplement: S2 Table — (PDF) [file pone.0231925.s002.pdf]

S2 Table. Reduced major axis (RMA) and phylogenetic RMA (PhyloRMA) regression of various parameters of the longest primary of 27 species (see S1 Table). Intercept, slope and R^2 values are provided for both RMA and PhyloRMA while 95% confidence intervals (95% CI) of both intercept and slope are shown only for RMA. Slope is the scaling exponent of the allometric log-log-relationship. H0 is the slope (scaling exponent) against which the observed slope has been tested for difference: * = significantly different ($P < 0.001$), $^{-}$ = $P > 0.09$, $^{--}$ $P > 0.3$.

**Model**	**H0**	**PhyloRMA**			**RMA**				
		**Intercept**	**Slope**	**R^2**	**Intercept**	**95% CI**	**Slope**	**95% CI**	**R^2**
log(growth-rate by mass) ~ log(calamus cross-sectional area)	1	2.898	1.168*	0.946	2.901	2.866 , 2.935	1.142*	1.078 , 1.211	0.981
log(growth-rate by mass) ~ log(calamus circumference)	2	1.609	2.336*	0.946	1.645	1.534 , 1.749	2.279*	2.151 , 2.415	0.981
log(growth-rate by length) ~ log(calamus cross-sectional area)	1	0.463	0.311*	0.584	0.523	0.491 , 0.549	0.236*	0.185 , 0.297	0.755
log(growth-rate by length) ~ log(calamus circumference)	1	0.121	0.621*	0.583	0.264	0.163 , 0.346	0.471*	0.369 , 0.594	0.754
log(calamus cross-sectional area) ~ log(feather-length)	2	-4.007	2.111 $^{-}$	0.967	-3.895	-4.176 , -3.631	2.053 $^{--}$	1.931 , 2.183	0.978
log(calamus circumference) ~ log(feather-length)	1	-1.451	1.055 $^{-}$	0.969	-1.401	-1.537 , -1.272	1.029 $^{--}$	0.969 , 1.092	0.979
log(growth-rate by mass) ~ log(feather-length)	3	-1.781	2.464*	0.946	-1.545	-1.985 , -1.139	2.344*	2.155 , 2.548	0.961
log(growth-rate by length) ~ log(feather-length)	0.5	-0.781	0.655*	0.598	-0.392	-0.673 , -0.163	0.482 $^{--}$	0.376 , 0.613	0.744
log(feather mass) ~ log(feather-length)	3	-4.236	2.912 $^{-}$	0.985	-4.222	-4.535 , -3.924	2.895 $^{-}$	2.756 , 3.041	0.986
log(feather mass) ~ log(body mass)	1	-0.422	1.095 $^{-}$	0.935	-0.176	-0.407 , 0.032	1.023 $^{--}$	0.926 , 1.131	0.944
log(feather-length) ~ log(body mass)	0.333	1.311	0.376 $^{-}$	0.885	1.395	1.274 , 1.499	0.355 $^{--}$	0.306 , 0.411	0.887
log(growth-rate by length) ~ log(body mass)	1	0.078	0.246*	0.522	0.279	0.171 , 0.364	0.172*	0.132 , 0.223	0.718
