# Supplementary material for: Determinants and constraints of feather growth
Source: PLoS One. 2020 Apr 24;15(4):e0231925. doi: 10.1371/journal.pone.0231925 (PMC7182269; doi:10.1371/journal.pone.0231925)
Supplement: S3 Table — (PDF) [file pone.0231925.s003.pdf]

S3 Table. Reduced major axis regressions of various parameters of the remiges (primaries and secondaries of Golden Eagles. Intercept and slope with their 95% confidence intervals (95% CI), as well as R^2 are shown. H0 is the slope (scaling exponent) against which the observed slope has been tested for difference: * = significantly different (P < 0.001), -- = P > 0.3..

**Model**	**H0**	**Intercept**	**95% CI**	**Slope**	**95% CI**	**R^2**
log(growth-rate by mass) ~ log(calamus cross-sectional area)	1	0.253	0.141 , 0.356	0.971 --	0.898 , 1.049	0.941
log(growth-rate by mass) ~ log(calamus circumference)	2	-0.791	-0.988 , -0.608	1.922 --	1.777 , 2.078	0.940
log(growth-rate by length) ~ log(calamus cross-sectional area)	1	-0.590	-0.726 , -0.481	0.316*	0.24 , 0.411	0.572
log(growth-rate by length) ~ log(calamus circumference)	1	-0.932	-1.171 , -0.741	0.627*	0.476 , 0.816	0.573
log(calamus cross-sectional area)~ log(feather-length)	2	-0.879	-1.104 , -0.673	1.492*	1.358 , 1.637	0.916
log(calamus circumference) ~ log(feather-length)	1	0.099	-0.015 , 0.204	0.753*	0.686 , 0.827	0.915
log(growth-rate by mass) ~ log(feather-length)	3	-0.600	-0.811 , -0.404	1.447*	1.321 , 1.584	0.920
log(growth-rate by length) ~ log(feather-length)	0.5	-0.853	-1.081 , -0.671	0.462 --	0.343 , 0.609	0.544
log(feather mass) ~ log(feather-length)	3	-2.847	-2.984 , -2.715	2.050*	1.965 , 2.139	0.981
