# Supplementary material for: Determinants and constraints of feather growth
Source: PLoS One. 2020 Apr 24;15(4):e0231925. doi: 10.1371/journal.pone.0231925 (PMC7182269; doi:10.1371/journal.pone.0231925)
Supplement: S4 Table — (PDF) [file pone.0231925.s004.pdf]

S4 Table. Linear mixed effects models applied to the parameters of Golden Eagle remiges. Intercept, *β*-coefficients for the two independent variables (with their significance; * $P < 0.0001$; -- $P > 0.67$), marginal R^2 (without the random effect individual) and conditional R^2 (with the random effect individual) is shown. Massiveness = mass of feather material per mm feather-length.

**Model**	**Intercept**	**β (slope)**	**R^2**	
			**Marginal**	**Conditional**
log(growth-rate by mass) ~ log(calamus cross-sectional area) + (feather type)	0.301	0.942*; -0.007--	0.935	0.949
log(growth-rate by mass) ~ log(calamus circumference) + (feather type)	-0.695	1.852*; -0.008--	0.935	0.947
log(growth-rate by length) ~ log(calamus cross-sectional area) + (feather type)	-0.676	0.357*; 0.042*	0.632	0.650
log(growth-rate by length) ~ log(calamus circumference) + (feather type)	-1.050	0.699*; 0.042*	0.630	0.643
log(growth-rate by length) ~ log(feather-length) + (feather type)	-1.192	0.649*; 0.073*	0.673	0.673
log(feather mass) ~ log(feather-length) + log(massiveness)	-0.001	0.999*; 0.999*	0.999	0.999
log(feather mass) ~ log(feather-length) + (feather type)	-2.561	1.879* ; -0.034*	0.974	0.988
