# Supplementary material for: Determinants and constraints of feather growth
Source: PLoS One. 2020 Apr 24;15(4):e0231925. doi: 10.1371/journal.pone.0231925 (PMC7182269; doi:10.1371/journal.pone.0231925)
Supplement: S5 Table — (PDF) [file pone.0231925.s005.pdf]

S5 Table. Reduced major axis regressions of various parameters of the primaries of 6 passerine species. Intercept and slope with their 95% confidence intervals (95% CI), as well as R^2 are shown. H0 is the slope (scaling exponent) against which the observed slope has been tested for difference: * = significantly different (P < 0.001), ¨ P > 0.67.

**Model**	**H0**	**Intercept**	**95% CI**	**Slope**	**95% CI**	**R^2**
log(growth-rate by mass) ~ log(calamus cross-sectional area)	1	-0.116	-0.129 , -0.104	1.046 ¨	0.929 , 1.181	0.842
log(growth-rate by mass) ~ log(calamus circumference)	2	-1.276	-1.437 , -1.136	2.092 ¨	1.86 , 2.357	0.845
log(growth-rate by length) ~ log(cross-sectional area of calamus)	1	0.471	0.443 , 0.487	0.229*	0.057 , 0.487	0.096
log(growth-rate by length) ~ log(calamus circumference)	1	0.213	-0.099 , 0.421	0.463*	0.122 , 0.978	0.099
log(cross-sectional area of calamus) ~ log(feather-length)	2	-3.721	-3.961 , -3.496	1.983 ¨	1.866 , 2.107	0.954
log(calamus circumference) ~ log(feather-length)	1	-1.306	-1.416 , -1.202	0.992 ¨	0.938 , 1.049	0.960
log(growth-rate by mass) ~ log(feather-length)	3	-4.011	-4.606 , -3.499	2.075*	1.811 , 2.384	0.801
log(growth-rate by length) ~ log(feather-length)	0.5	-0.329	-1.245 , 0.312	0.426*	0.094 , 0.901	0.093
log(feather mass) ~ log(feather-length)	3	-3.721	-3.981 , -3.474	2.669*	2.541 , 2.804	0.969
