# Supplementary material for: Determinants and constraints of feather growth
Source: PLoS One. 2020 Apr 24;15(4):e0231925. doi: 10.1371/journal.pone.0231925 (PMC7182269; doi:10.1371/journal.pone.0231925)
Supplement: S6 Table — (PDF) [file pone.0231925.s006.pdf]

S6 Table. Linear mixed effects models applied to the parameters of the primaries of 6 passerine species. Intercept, *β*-coefficients for the two or three independent variables (with their significance; * P < 0.0001; ¨ P > 0.67), marginal R^2 (without the random effect individual) and conditional R^2 (with the random effect individual) is shown. Massiveness = mass of feather material per mm feather-length.

Model	Intercept	β (slope)	R^2	
			Marginal	Conditional
log(growth-rate by mass) ~ log(calamus cross-sectional area) + (wingtip primary) [1]	-0.087	0.964* ; -0.067*	0.833	0.988
log(growth-rate by mass) ~ log(calamus circumference) + (wingtip primary) [1]	-1.159	1.929* ; -0.061*	0.833	0.989
log(growth-rate by length) ~ log(calamus cross-sectional area) + (wingtip primary) [1]	0.494	0.224* ; -0.072*	0.302	0.958
log(growth-rate by length) ~ log(calamus circumference) + (wingtip primary) [1]	0.240	0.456* ; -0.071*	0.308	0.961
log(calamus cross-sectional area) ~ log(feather-length) + (wingtip primary) [1]	-2.841	1.518* ; 0.044*	0.872	0.978
log(calamus cross-sectional area) ~ log(feather-length) + log(massiveness) + (wingtip primary) [1]	-0.561	0.557* ; 0.827*; 0.013	0.979	0.991
log(growth-rate by mass) ~ log(feather-length)+ log(massiveness) + (wingtip primary) + (1|species) [1]	-0.685*	0.575* ; 0.826* ; -0.057*	0.879	0.995
log(growth-rate by length) ~ log(feather-length)+ (wingtip primary) + (1|species)*	-0.214*	0.377* ; -0.063*	0.249	0.963
logFeathermass ~ logFeatherlength + logMassperlength	-0.001	0.999* ; 0.999*	0.999	0.999
logFeathermass ~ logFeatherlength + Wingtip	-2.721	2.143* ; 0.037*	0.908	0.994

[1] Species as random intercept effect is highly significant at p < 0.001.
