# Supplementary material for: Determinants and constraints of feather growth
Source: PLoS One. 2020 Apr 24;15(4):e0231925. doi: 10.1371/journal.pone.0231925 (PMC7182269; doi:10.1371/journal.pone.0231925)
Supplement: Attachment — Submitted filename: Response to Reviewers.docx [file pone.0231925.s007.docx]

**Response to Reviewers**

We thank the reviewers for their positive assessment of our manuscript. Here is how we dealt with the points raised:

Reviewer #1: Though I have used feather growth rate as a dependent variable to study life history questions, I have not considered the physiology behind feather growth in the depth presented in this paper. The great strength of the paper is how they examined hypotheses about the control of feather growth from three perspectives. They tested feather growth concepts across species by holding feather ID constant and in choosing the longest primary as the focal feather, they chose the feather whose growth may be the rate limiting step in moult. They also held species constant (golden eagle) and looked at growth across feather types. Finally, they varied feather size while holding feather type constant (all the primaries) to look at what controls growth rate. I was happy with their statistical choices which took into account the non-independence of closely related species. In fact, their explanation of statistical procedures were worthy of a stats textbook.

I have no real issues with regard to publication. It may be, in part, because this is not my field. A minor complaint is that in the cross-species analysis there were a few species represented by only one individual. At this point, that is not worth pursuing. This paper would be of (slightly) wider interest if the authors discussed their results relative to the utility of using growth bars (mm/d) as an index of feather growth vs. mass/d. Everyone who uses ptilochronology as a technique would want to read this paper.

The utility of using growth bars (mm/d) as an index of feather growth vs. mass/d willl be fully treated and put into context in the book “The Biology of Moult in Birds” to be published in July 2020.

The study well-conceived and executed. It was a creative and thorough test of their hypotheses. The acknowledgements thanked “Robert A. Robinson for revising and improving the English.” He did a good job.

Reviewer #2: In this study, Jenni et al. examine the factors that influence feather growth in birds. By using three separate datasets, the authors clearly demonstrate that follicle size influences feather growth rate by mass, as well as structural design. In other words, the feather follicle size, which the authors estimate by measuring the cross-sectional size of the feather calamus, determines the amount of feather produced during a unit of time, then used in the production of a feather, which varies in massiveness (mass/length). These results help explain the observation that larger feathers need disproportionately longer to grow than small feathers.
Though growth rates in this study are based on estimates from the literature, as well as direct measurements, the results are robust. The use of three distinct datasets allows for thorough examination of within-individual, intraspecific, and interspecific variation. The analyses appear sound and I was happy to see that the authors included phylogenetic regressions and that those results seem to indicate that phylogenetic effects on these relationships are minimal.
Overall my comments are relatively minor. There are some issues with wording and clarity throughout. I’ve attempted to highlight a number of these spots. In addition, though the conclusions section was clear and well-written, the manuscript would benefit from additional text on implications.

Line 36: “two-dimensional parameter” isn’t clear to the reader and requires explanation or re-wording.

It is true that “two-dimensional parameter” is quite an abstract expression. It is derived from the finding that it was not the circumference (one-dimensional), but the cross-sectional area (two-dimensional) which correlated directly with feather-growth. We now changed it to “factors acting at a two-dimensional scale”.

Line 37: “linear (circular)” reads as an oxymoron. Though this description is made clear later on in the text, it’s confusing in the abstract.

We do not think that this is an oxymoron, because a circle is a line, while a disc would refer to the circle plus its content (hence a two-dimensional surface), although colloquially a circle is often (mostly) understood as a two-dimensional thing. We now rephrased it as “the linear arrangement of stem cells (in a circular configuration)”, also on line 346. Rohwer et al (2009) also use similar expressions.

Line 38: missing word in “, hence seems”

Done

Line 52: cut “for many reasons”

Done

Line 57: suggest rewording to “, like cornified structures such as claws and hair”

Done

Line 59: logic disagreement with “dropped” and the rest of the sentence. A dropped feather cannot be fragile or vulnerable

Rephrased

Line 66. Cite the first sentence.

Done

Line 71: change “a little” to “slightly”

Done

Line 89: cut “so”

Done

Line 94: “ramogenic” should be defined.

We feel that the explanation, what the ramegenic zone is, is given immediately after the expression “ramogenic zone” in the sub-clause “where differentiation, elongation and keratinization of the cells occurs”.

Line 119-120: cut sentence.

We think that it is convenient for the reader to be told that the type of feather is the same (in contrast to the other two datasets which follow). Therefore, we would like to keep this sentence.

Line 121-122: I’m not sure it makes sense to define a theoretical minimum time of moult if all feathers are never moulted concurrently.

There is a series of species which do moult all wing-feathers concurrently.

Line 270: “although it had in a” – awkward wording.

We changed it to “although there was an effect in a”

Line 328-329: award wording, suggest rephrasing.

We split it now in two sentences.

Line 347: two-dimensional scale still isn’t made clear to the reader.

As explained above, we derived this from the finding that it was not the circumference, but the cross-sectional area which correlated directly with feather-growth. However, which factors these may be can only be guessed and, therefore, we feel unable to be more precise.

Line 423: suggest different word than “acceptable”

We changed to “was still significant”.

Line 438: cut or reword “by far”

Done

Fig. 5: Probably not necessary. If kept, needs to be modified.

We agree that this figure can also be omitted. However, colleagues who read the mansucript were very happy about this concise graphical summary. Therefore, we would like to keep it, but leave it to the editor to decide. We do not really understand why and how this graph “needs to be modified”.
